# Dr. Robert W. Green

**Published:** 1908-12

**Authors:** 


DR. ROBERT W. GREEN, of Geneseo, N. Y., died at his home October 5, 1908. of angina pectoris, aged 64 years. He was a member of the Medical Society of the State of New York, a Civil War Veteran, a former health officer of his village, also trustee of Geneseo, county physician, and member of the board of education. He graduated from the Medical Department, University of Buffalo in 1889.

